# dbPAF: an integrative database of protein phosphorylation in animals and fungi

**DOI:** 10.1038/srep23534

**Published:** 2016-03-24

**Authors:** Shahid Ullah, Shaofeng Lin, Yang Xu, Wankun Deng, Lili Ma, Ying Zhang, Zexian Liu, Yu Xue

**Affiliations:** 1 Department of Bioinformatics & Systems Biology, College of Life Science and Technology, Huazhong University of Science and Technology, Wuhan, Hubei 430074, China; 2 Key Laboratory of Molecular Biophysics of the Ministry of Education, College of Life Science and Technology, Huazhong University of Science and Technology, Wuhan, Hubei 430074, China

## Abstract

Protein phosphorylation is one of the most important post-translational modifications (PTMs) and regulates a broad spectrum of biological processes. Recent progresses in phosphoproteomic identifications have generated a flood of phosphorylation sites, while the integration of these sites is an urgent need. In this work, we developed a curated database of dbPAF, containing known phosphorylation sites in *H. sapiens*, *M. musculus*, *R. norvegicus*, *D. melanogaster*, *C. elegans*, *S. pombe* and *S. cerevisiae*. From the scientific literature and public databases, we totally collected and integrated 54,148 phosphoproteins with 483,001 phosphorylation sites. Multiple options were provided for accessing the data, while original references and other annotations were also present for each phosphoprotein. Based on the new data set, we computationally detected significantly over-represented sequence motifs around phosphorylation sites, predicted potential kinases that are responsible for the modification of collected phospho-sites, and evolutionarily analyzed phosphorylation conservation states across different species. Besides to be largely consistent with previous reports, our results also proposed new features of phospho-regulation. Taken together, our database can be useful for further analyses of protein phosphorylation in human and other model organisms. The dbPAF database was implemented in PHP + MySQL and freely available at http://dbpaf.biocuckoo.org.

As one of the most well-studied and ubiquitous post-translational modifications (PTMs) in proteins, phosphorylation is a conserved mechanism in both eukaryotes and prokaryotes, participates in almost all of biological processes, and reversibly determines the cellular dynamics and plasticity^1^,^2^,^3^,^4^,^5^. In eukaryotes, phosphorylation mainly occurs on a subset of three types of amino acids, including serine, threonine and tyrosine residues. Phosphorylation events are dynamically but precisely regulated, and the core machinery of a phosphorylation system contains numerous protein kinases (PKs) for modifying proteins as “writers”, phospho-binding proteins for recognizing and interacting with phosphorylation sites as “readers”, and protein phosphatases (PPs) for demodifying substrates as “erasers”^2^,^3^,^6^,^7^. The aberrant phosphorylation has been extensively reported to be highly associated with human diseases, such as cancers^8^, neurodegenerative diseases^9^ and metabolic disorders^10^. In this regard, the identification and functional analysis of phosphosites are fundamental for understanding the molecular mechanisms and regulatory roles of protein phosphorylation.

Recently, rapid progresses in high-throughput liquid chromatography/mass spectrometry (LC-MS) techniques have greatly advanced the identification of phosphorylation sites^11^,^12^,^13^. For example, using a mouse model of multistage skin carcinogenesis, Zanivan *et al*. identified and quantified 3,457 proteins with 5,249 phosphorylation sites^12^. Also, using a label-free technology, Lundby *et al*. totally quantified 31,480 phosphorylation sites in 7,280 proteins across 14 rat organs and tissues^13^. Recently, Humphrey *et al*. developed a new platform of EasyPhos, which can rapidly quantify over 10,000 phosphorylation sites from cell or tissue samples in a single run^11^. Since a flood of sites have been generated, computational analysis of the phosphoproteomic data in a systematic level has also been boomed, such as the prediction of site-specific kinase-substrate relations (ssKSRs)^14^,^15^,^16^, the re-construction and modeling of phosphorylation networks^1^,^17^, the molecular evolutionary analysis of phosphorylation signalings^6^,^18^, and the identification of genetic variations that potentially change phosphorylation^19^,^20^. Thus, we can expect that more and more experimental and computational studies will be carried out in the near future, to accumulatively enhance our knowledge on the phosphorylation.

Due to the data accumulation, the collection and integration of numerous phosphorylated substrates with their sites from different studies have emerged to be a great challenge. The first phosphorylation database, PhosphoBase, was developed in 1998 and only contained 398 experimentally identified phosphorylation sites at that time^21^. In 2004, this database was merged into a new database of Phospho.ELM^22^,^23^, and its 9.0 version contained 41,070 phosphorylation sites in 10,601 substrates (Table 1). In 2006, two phosphorylation databases of dbPTM^24^ and PHOSIDA^25^ were released, and both databases were continuously maintained nearly ten years until now^26^,^27^. To date, one of the most comprehensive and popular database is PhosphositePlus, which was initially established in 2004 and currently contained 310,779 phosphosites of 37,568 proteins^28^,^29^ (Table 1). In contrast to a general collection of phosphosites, several databases were mainly focused on specific species for a better annotation, because the number of known phosphorylation sites is still too limited in most of organisms. For example, PhosphoPep only maintained phosphorylation sites for 4 model organisms, including *H. sapiens*, *D. melanogaster*, *C. elegans* and *S. cerevisiae*^30^,^31^, while PhosphoGRID only collected known phosphosites in *S. cerevisiae*^32^,^33^. PHOSIDA was also organized in a species-specific manner, containing phosphorylation sites in nine prokaryotic or eukaryotic organisms^25^,^26^. In addition, although several databases, such as SysPTM^34^,^35^, HPRD^36^ and UniProt^37^ were constructed for a more general purpose, they also maintained a large number of phosphorylation sites.

During the past decade, we also collected and curated phosphorylation sites beyond directly using known data sets from public databases. Previously, we developed two phosphorylation databases of dbPPT^5^ and dbPSP^4^ for plants and prokaryotes, respectively. Here we reported a new database of dbPAF for the phospho-sites in human, animals and fungi. From the scientific literature and public databases, we totally collected and integrated 483,001 known phosphorylation sites in 54,148 proteins from *H. sapiens*, *M. musculus*, *R. norvegicus*, *D. melanogaster*, *C. elegans*, *S. pombe* and *S. cerevisiae*. The detailed annotations together with original references were provided for each protein entry. Using the new data set, we detected significantly over-represented sequence motifs around phospho-serine (*p*S), phospho-threonine (*p*T) and phospho-tyrosine (*p*Y) sites for each species, separately. From the results, we observed that the most significant *p*T motifs are highly conserved across different organisms, while *p*S sites prefer to occur at intrinsically disordered regions with low-complexity. We also predicted potential PKs for phospho-sites and demonstrated that different PK groups or families play different roles in the regulation of phosphorylation. In addition, we systematically analyzed the conservation states of phosphorylation sites, and observed that the phosphorylation is similarly conserved across different species. Taken together, the dbPAF database can serve as a useful resource for further studies of protein phosphorylation in human, animals and fungi.

## Results

### Database construction and data statistics

**Figure 1:**
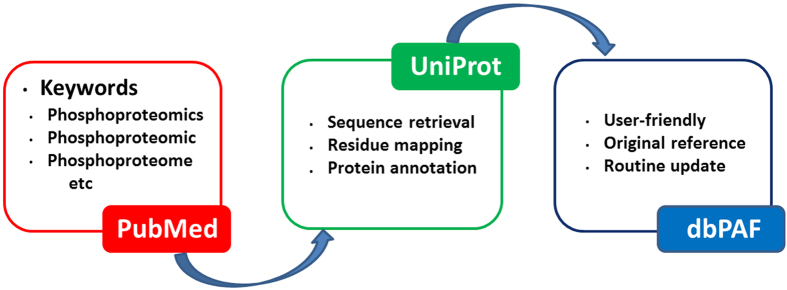
The procedure for the construction of dbPAF database. Also, we also integrated know phosphorylation sites from several public databases, including Phospho.ELM^22^,^23^, dbPTM^24^,^27^, PHOSIDA^25^,^26^, PhosphositePlus^28^,^29^, PhosphoPep^30^,^31^, PhosphoGRID^32^,^33^, SysPTM^34^,^35^, HPRD^36^ and UniProt^37^.

In this work, we mainly focused on the collection and integration of known phosphosites identified from large-scale phosphoproteomic studies, and the procedure for the implementation of dbPAF database was shown in Fig. 1. First, we searched the PubMed with multiple keywords, including “phosphoproteomics”, “phosphoproteomic” and “phosphoproteome”. All retrieved articles were carefully curated, and we directly took the identified phosphorylated proteins, peptides and sites from the supplementary materials published together with these manuscripts if available. Because only a handful of eukaryotic species had the enough phosphorylation information, here we only reserved phosphorylation sites in *H. sapiens*, *M. musculus*, *R. norvegicus*, *D. melanogaster*, *C. elegans*, *S. pombe* and *S. cerevisiae*. Totally, we collected 294,370 non-redundant phosphorylation sites of 40,432 proteins from 115 published papers (Supplementary Table S1). For each species, we mapped corresponding phosphorylated proteins to its proteome sequences downloaded from UniProt database^37^, and the phosphorylation sites were exactly pinpointed (Fig. 1). The detailed annotations such as protein names, gene names, keywords, functional descriptions and sequence annotations of phosphoproteins were retrieved from UniProt and further integrated, while the original references of identified phosphorylation sites were also provided in dbPAF (Fig. 1).

Besides the manual curation of the literature, we also integrated known phosphorylation sites of the seven species from several public databases, including Phospho.ELM^22^,^23^, dbPTM^24^,^27^, PHOSIDA^25^,^26^ , PhosphositePlus^28^,^29^ , PhosphoPep^30^,^31^, PhosphoGRID^32^,^33^, SysPTM^34^,^35^, HPRD^36^ and UniProt^37^ (Table 1). For multiple entries with an identical phosphosite in the same protein, only one record was reserved. Finally, dbPAF contained 483,001 known phosphorylation sites of 54,148 proteins, as a comprehensive data resource for human, animals and fungi.

**Figure 2:**
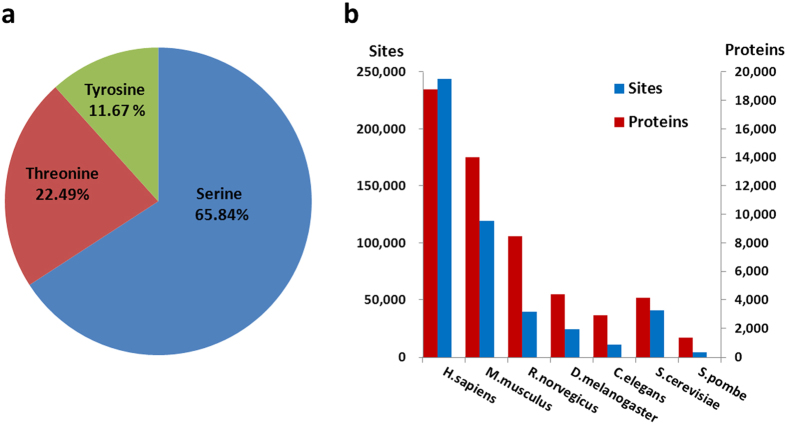
The data statistics of dbPAF. (**a**) The distribution of *p*S, *p*T and *p*T residues. (**b**) The distribution of phosphorylated proteins and sites in each species.

With the data set, we counted the distribution of phospho-sites for different amino acid types, and observed that the phosphorylation predominantly modifies *p*S residues (65.84%), while only 22.49% and 11.67% of phosphorylation events occur in *p*T and *p*Y sites, respectively (Fig. 2a). At the current stage, the phosphorylation events are not equally profiled in each species, whereas 50.52% (244,034 sites in 18,773 proteins) and 24.71% (119,328 sites in 14,044 proteins) of total phosphorylation sites were detected in *H. sapiens* and *M. musculus*, separately (Fig. 2b). In contrast, only 1,389 phosphorylated proteins with 3,957 sites were identified in *S. pombe*.

### Usage of the dbPAF database

**Figure 3:**
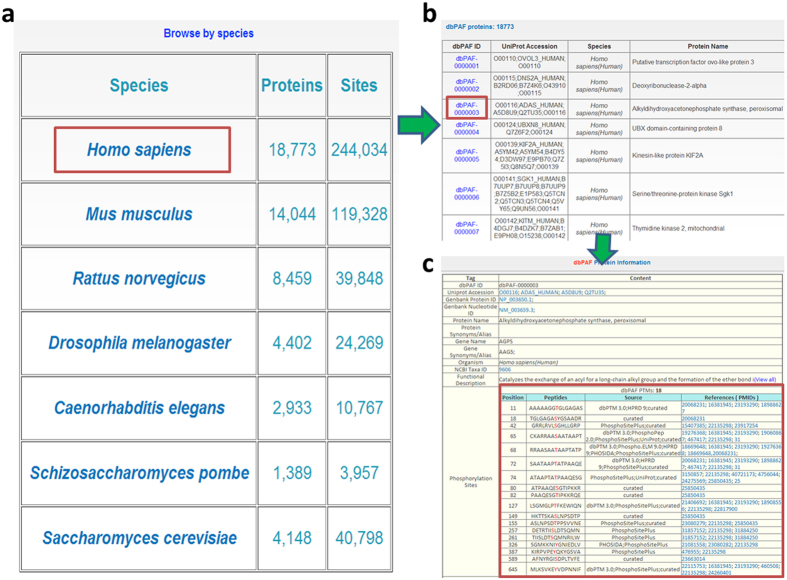
The “ Browse by species” option. (**a**) Phosphorylated proteins can be browsed in a species-specific manner by clicking on the corresponding diagram. (**b**) The phosphorylated substrates will be listed in a tabular format. (**c**) The detailed annotations of human AGPS together with known phospho-sites.

Our database was developed in an easy-to-use manner, and multiple options were provided to access the phosphorylation information. First, phosphorylated substrates in dbPAF can be browsed in a species-specific mode (Fig. 3a). Here we chose human peroxisomal alkyldihydroxyacetonephosphate synthase (AGPS) as an example to demonstrate the usage of “Browse by species”. After clicking on the species diagram of *H. sapiens* (Fig. 3a), all human phosphorylated proteins would be listed in a tabular format with “dbPAF ID”, “UniProt Accession”, “Species”, and “Protein Name” (Fig. 3b). A unique “dbPAF ID” was assigned to each protein for the convenient organization of dbPAF database. Then by clicking on the “dbPAF-0000003”, the detailed annotations including 18 known phospho-sites of human AGPS could be shown (Fig. 3c).

**Figure 4:**
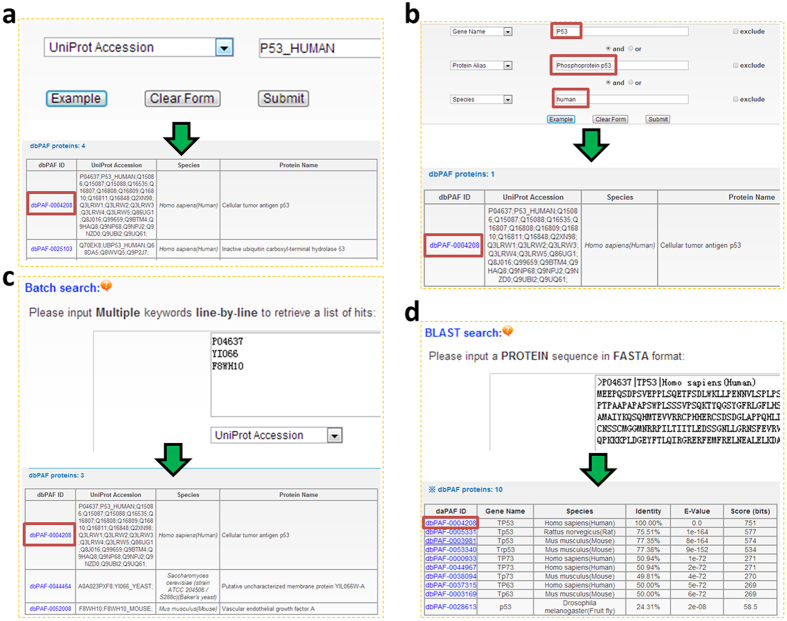
The search options. (**a**) “Substrate Search” with one or multiple keywords. (**b**) The “Advanced search” permitted users to input up to three terms for query. (**c**) The “Batch Search” for retrieveing multiple protein entries with a list of terms. (**d**) The database can be searched with a protein sequence in FASTA format to find identical or homolgous phosphoproteins.

Besides the “Browse by species” option, our database provided up to four search options including “Substrate Search” (Fig. 4a), “Advanced search” (Fig. 4b), “Batch Search” (Fig. 4c), and “Blast Search” (Fig. 4d). For the “Substrate Search”, users can input one or multiple keywords, e.g, using “P53_HUMAN” and selecting “UniProt Accession”, to find the phosphorylation information of human p53 (Fig. 4a). Also, users can choose the ‘Advanced Search’ using three terms together with three operators of “and”, “or” and “exclude”, to obtain a more exact hit (Fig. 4b). Moreover, “Batch Search” was present for retrieving multiple phosphoproteins with a list of keywords (Fig. 4c). Finally, “Blast Search” was implemented to find identical or homologous proteins using a protein sequence in FASTA Format. The NCBI BLAST package^38^ was adopted for the sequence alignment (Fig. 4d).

### The sequence preferences around phosphorylation sites

**Figure 5:**
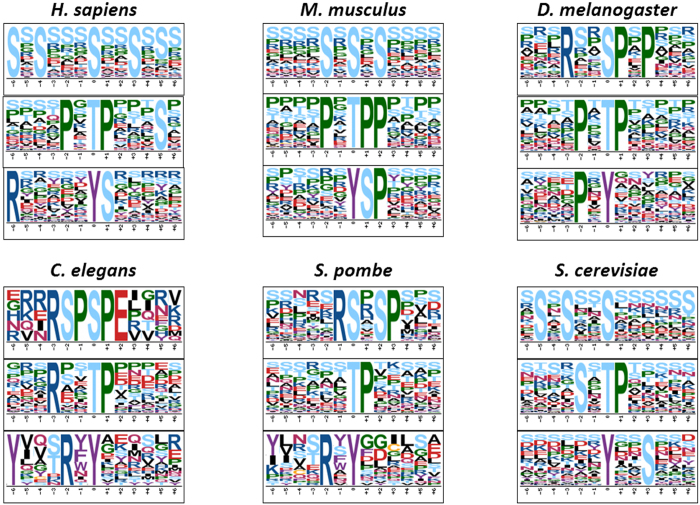
Motif-based analysis of sequence preferences around phosphorylation sites in *H. sapiens*, *M. musculus*, *D. melanogaster*, *C. elegans*, *S. pombe* and *S. cerevisiae*. In each species, the most significant motif was visualized for each type of phosphorylated residue. In the default threshold, we did not detect any *p*Y motifs for *S. pombe*, due to the data limitation. However, when we slightly relaxed the stringency, a significant *p*Y motif was detected, with a *p*-value <0.00001.

Previous studies demonstrated that short linear motifs around phorphorylation sites confer major specificity for the recognition, although a variety of cellular contextual factors, such as subcellular co-localization of PKs with their substrates, co-complex, or physical interaction, provide additional phosphorylation specificity *in vivo*^1^,^7^,^15^. Using the data set in dbPAF, here we performed a motif-based analysis of sequence preferences around phosphorylation sites in *H. sapiens*, *M. musculus*, *D. melanogaster*, *C. elegans*, *S. pombe* and *S. cerevisiae* (Fig. 5). The motifs in *R. norvegicus* was not computationally detected, because human and mouse can be representative for mammalians. In the results, the most significant motifs of *p*Y sites are quite different across different organisms, although the motif *p*YS in *H. sapiens* is considerably similar with *p*YSP in *M. musculus* (Fig. 5). A simple interpretation is that tyrosine phosphorylation was evolved in metazoans^39^, and there are no tyrosine kinases encoded in *S. pombe* and *S. cerevisiae*^40^. Thus, tyrosine phosphorylation in yeasts might be mediated by dual-specificity protein kinases which can phosphorylate both serine/threonine and tyrosine sites^41^. Also, during the evolution, the number of tyrosine kinases was significantly increased. For example, there are only 32 tyrosine kinases in *D. melanogaster*, but human has up to 90 tyrosine kinases^40^. Thus, different complexities of tyrosine phosphorylation in different species determine the distinct motif patterns. In contrast, the most significant motifs of *p*T sites in different species are quite similar (Fig. 5), and the results demonstrated a conserved mechanism for the threonine phosphorylation during evolution. Additionally, it was demonstrated that the phosphorylation sites preferentially occur in intrinsically disordered regions with low-complexity^42^. However, only most significant motifs of *p*S sites in *H. sapiens*, *M. musculus*, and *S. cerevisiae* follow the intrinsically disordered sequences, whereas *p*S motifs in other species are still informative (Fig. 5).

**Figure 6:**
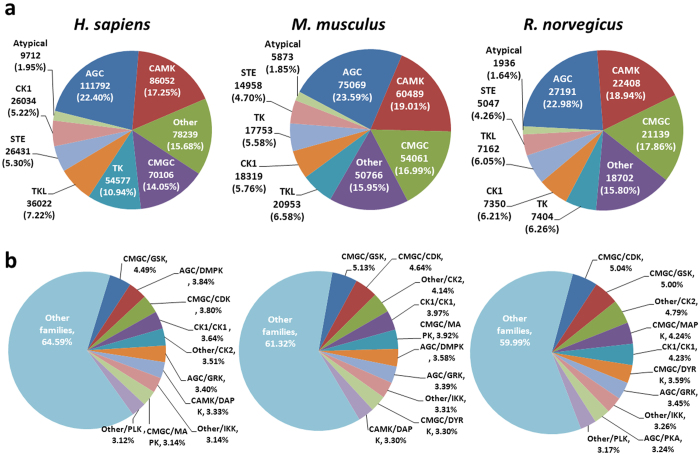
The distrubtions of PKs that were predicted to modify phospho-sites in *H. sapiens*, *M. musculus* and *R. norvegicus*, (**a**) in the kinase group level, and (**b**) in the kinase family level.

To further dissect sequence preferences of mammalian phosphorylation, we systematically predicted potential PKs for phosphorylation sites in *H. sapiens*, *M. musculus* and *R. norvegicus* (Fig. 6). A previously developed tool of GPS 2.1^16^ was used to predict ssKSRs in the family level (Fig. 6b) and then counted in the group level (Fig. 6a). In the kinase group level, the top four groups are AGC, CAMK, Other and CMGC kinase groups, which are responsible for the modification of about 70~75% of total phosphorylation events (Fig. 6a). In the family level, the top ten kinase families carry out about ~35% to 40% of total phosphorylation events (Fig. 6b). Thus, our results demonstrated that different kinase groups or families play distinct roles in mammalians, and combinatorially determine the phosphorylation preferences.

### The conservation distributions of phosphorylation sites

In a previous study, Minguez *et al*. developed the Residue Conservation Score (RCS) to determine the conservation status of PTM sites^43^. Because the overall conservation states of different proteins are not equal, they further used non-modified residues as a reference data set to normalize RCS and further calculated the relative RCS (rRCS) value for each PTM site. In their analysis, they totally collected ~93,000 phosphorylation sites, including 58,501, 20,880, 1,748, 1,951, 1,337 and 9,764 sites in *H. sapiens*, *M. musculus*, *R. norvegicus*, *D. melanogaster*, *C. elegans* and *S. cerevisiae*, respectively. Based on the data set, the average rRCS values were calculated as about 55~85%, exhibiting a strong difference across eukaryotes^43^.

**Figure 7:**
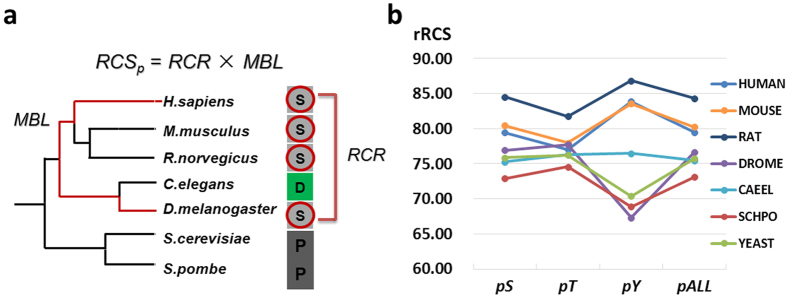
The evolutionary analysis of phosphorylation conservation. (**a**) As previously described^43^, *RCS*_*p*_ was calculated as RCR*MBL. (**b**) We further calculated rRCS values for phosphorylation sites, and the average rRCS values of *p*S, *p*T, *p*Y and all sites were shown for each species.

Here, with the newly integrated data set in dbPAF, we adopted the same procedure and re-performed the analysis of phosphorylation conservation in seven organisms (Fig. 7a). In contrast with the previously study^43^, our average rRCS values ranged from 73.10% (in *S. pombe*) to 84.34% (in *R. norvegicus*), and there was not a significant difference across different organisms (Fig. 7b, Table 2). For example, the average rRCS values are about 55~60% in *D. melanogaster*, *C. elegans* and *S. cerevisiae*, while our results are 76.65%, 75.50% and 75.72%, respectively (Fig. 7b). However, our average rRCS values on *H. sapiens* and *M. musculus* are similar with the previous study (Table 2). Thus, the previous results might be biased for lower species due to the data limitation^43^. When the data set was enlarged, each species shows a similar conservation status of phosphorylation. Also, for the *p*Y conservation, we observed that the rRCS values in mammalians are much higher, while the ones in lower species are <80% (Fig. 7b). Again, this is because the complexity of tyrosine phosphorylation regulation is much higher in mammalians with more tyrosine kinases^39^,^40^, and thus mammalian tyrosine phospho-sites undergo a stronger functional constraint during the evolution.

## Discussion

Recent progresses in the development and improvement of high-throughput phosphoproteomic techniques have facilitated a rapid increase of the number of identified phosphorylated proteins and sites in animals, fungi, plants^5^ and prokaryotes^4^. Due to the data accumulation, computational analysis of the big data has also emerged to be an intriguing topic, in contrast with conventionally experimental assays^1^,^6^,^14^,^15^,^16^,^17^,^18^,^19^,^20^. All of these studies are heavily dependent on a high quality data resource of phospho-sites. Although a number of public databases were developed^22^,^23^,^24^,^25^,^26^,^27^,^28^,^29^,^30^,^31^,^32^,^33^,^34^,^35^,^36^,^37^, no one can collect and maintain all known phospho-sites. Also, several databases were developed for a more general purpose. For example, SysPTM 2.0 contained modification sites for 50 types of PTMs across 2031 species^34^,^35^, whereas HPRD is one of the most useful resource for human proteins but not limited to PTMs^36^. UniProt is a popular database for protein annotations, and PTMs are just one part of features^37^. In addition, a considerable proportion of databases were developed for a general collection of phosphorylation sites, while only PhosphoPep^30^,^31^, PhosphoGRID^32^,^33^ and PHOSIDA^25^,^26^ were constructed for specific organisms with a rich data set, since few phospho-sites were reported in most of species. Thus, the collection, integration, and annotation of phosphorylation sites in an organism-specific manner can be highly useful for further studies of phosphorylation in a specific species.

In this work, we reported a new database of dbPAF with 483,001 known phosphorylation sites in 54,148 proteins from human, animals and fungi. By comparison, our database contained more phospho-sites than other databases (Table 1). Because phosphorylated proteins, peptides and sites from different papers or databases might be differentially processed and annotated with distinct criteria, it’s a great challenge to ensure the standardization of data quality. Although several pilot studies have been performed for the quality control of phosphoproteomic data sets, the experimental evidences, e.g., the use of phospho-specific antibodies to verify the existence of normalized phosphosites^44^, yet remain to be provided. Because this study was mainly focused on the collection and integration of known phosphosites, any further normalization or standardization of the data set was not carried out. Based on the newly integrated data set, we carefully analyzed the sequence motifs around *p*S, *p*T and *p*Y sites (Fig. 5), predicted potential PKs that are responsible for modifying the phosphorylation sites, and performed an analysis of the phosphorylation conservation status across different organisms. We anticipated that such a database can be a useful resource for further analyses. The database will be continuously updated and maintained when new phosphorylation sites are reported.

## Methods

### Motif-based analysis of phospho-sites

In this study, we chose Motif-X (http://motif-x.med.harvard.edu/motif-x.html), a widely-used online tool for detecting phosphorylation motifs from the phosphoproteomic data^45^. For each species, known phosphorylated peptides in length of 13 with central characters of S/T/Y residues were prepared as the foreground data set, while non-phosphorylated peptides in the same proteins were regarded as the background data set. The default parameters were adopted, with a *p*-value <0.000001. The phosphorylation motifs were calculated for *p*S, *p*T and *p*Y sites, respectively. The most significant motifs of the three types of residues for each species were diagrammed in sequence logos (Fig. 5).

### Prediction of kinase-specific phosphorylation sites in the kinase family level

Previously, we developed a software package of GPS 2.1^16^, in which the predictors were established based on the kinase classifications of mammalians. Thus, here we only predicted potentially kinase-specific phospho-sites in *H. sapiens*, *M. musculus* and *R. norvegicuse*, respectively. Also, because the prediction accuracy at the group level is limited, here we only predicted ssKSRs for known phosphorylation sites in the family level, and further counted in the group level. Totally, 42 and 20 predictors were selected from GPS 2.1 for serine/threonine kinases and tyrosine kinases, separately. The high thresholds were adopted for the prediction.

### The calculation of RCS and rRCS

We analyzed the conservation distributions of phosphorylation sites in *H. sapiens*, *M. musculus*, *R. norvegicus*, *D. melanogaster*, *C. elegans*, *S. pombe* and *S. cerevisiae*, separately. First, the InParanoid 4.1 program^46^ was obtained from Stockholm Bioinformatics Centre (http://InParanoid.sbc.su.se) for pairwisely detecting orthologs among the seven species. All orthologous proteins among different species were multi-aligned by Clustal Omega (http://www.clustal.org/omega/)^47^. As previously described^43^, for each species, if a serine, threonine, or tyrosine residue in a multiple sequence alignment (MSA) is phosphorylated, the corresponding column was regarded as the modified position. Other columns containing serines, threonines or tyrosines were taken as the reference positions. To calculate the conservation of a phosphorylated site from a MSA, we adopted a previously defined *RCS*_*p*_^43^, which can be calculated as below:


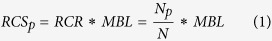


The *N*_*p*_/*N* was defined as the Residue Conservation Ratio (*RCR*)^43^. The *N* is the number of sequences with the maximum branch length (*MBL*), which is the maximum branch distance between any two species that contain a conserved phospho-site. The *N*_*p*_ is the number of phospho-sites observed in the column. Then the calculated *RCS*_*p*_ values of phosphorylated sites were mapped into the reference distribution to calculate the relative *RCS* or *rRCS*^43^. The species tree of the seven organisms was taken from the Interactive Tree Of Life (iTOL, http://itol.embl.de/)^48^.

## Additional Information

**How to cite this article**: Ullah, S. *et al*. dbPAF: an integrative database of protein phosphorylation in animals and fungi. *Sci. Rep*. **6**, 23534; doi: 10.1038/srep23534 (2016).

## Supplementary Material

Supplementary Information

## Figures and Tables

**Table 1:** The comparison of dbPAF with other public databases.

Database	Proteins	Sites	*p*S	*p*T	*p*Y
dbPAF	54148	483001	318016	108615	56370
Phospho.ELM 9.0	10601	41070	30653	7232	3185
dbPTM 3.0	24601	147851	102310	28797	16744
PHOSIDA	15924	64118	51318	10782	2018
PhosphositePlus	37568	310779	194217	69647	46915
PhosphoPep 2.0	16234	75278	57762	13492	4024
PhosphoGRID 2.0	3121	19831	14871	4343	617
SysPTM 2.0	13867	54224	41875	9126	3223
HPRD 9	8280	51733	36052	11388	4293
UniProt^*a*^	14904	50713	42189	6943	1581

^*a*^For the UniProt database, only experimentally verified phospho-sites were considered, whereas the sites annotated with “By similarity”, “Potential” or “Probable” were not included.

**Table 2:** For each species, the conservation distribution were calculated for *p*S, *p*T and *p*Y residues, respectively.

Organism	*p*S		*p*T		*p*Y		Final rRCS^*c*^
	RCS^*a*^	rRCS^*b*^	RCS	rRCS	RCS	rRCS	
*H. sapiens*	0.19	79.44	0.19	77.04	0.23	83.90	79.53
*M. musculus*	0.20	80.48	0.20	78.00	0.26	83.61	80.26
*R. norvegicus*	0.19	84.56	0.20	81.76	0.22	86.85	84.34
*D. melanogaster*	0.17	76.97	0.17	77.75	0.20	67.32	76.65
*C. elegans*	0.22	75.27	0.21	76.32	0.30	76.48	75.50
*S. pombe*	0.22	72.92	0.23	74.60	0.30	68.90	73.10
*S. cerevisiae*	0.19	75.91	0.19	76.23	0.24	70.38	75.72

^*a*^The avarage RCS value; ^*b*^The avarage rRCS value; ^*c*^The final rRCS value.

## References

[b1] Linding R. . Systematic discovery of *in vivo* phosphorylation networks. Cell 129, 1415–1426 (2007). 17570479 10.1016/j.cell.2007.05.052 PMC2692296

[b2] Jin J. & Pawson T. Modular evolution of phosphorylation-based signalling systems. Philos Trans R Soc Lond B Biol Sci 367, 2540–2555 (2012). 22889906 10.1098/rstb.2012.0106 PMC3415845

[b3] Seet B. T., Dikic I., Zhou M. M. & Pawson T. Reading protein modifications with interaction domains. Nat Rev Mol Cell Biol 7, 473–483 (2006). 16829979 10.1038/nrm1960

[b4] Pan Z. . dbPSP: a curated database for protein phosphorylation sites in prokaryotes. Database (Oxford) 2015, bav031 (2015). 25841437 10.1093/database/bav031 PMC4385273

[b5] Cheng H. . dbPPT: a comprehensive database of protein phosphorylation in plants. Database (Oxford) 2014, bau121 (2014). 25534750 10.1093/database/bau121 PMC4273206

[b6] Li L. . The human phosphotyrosine signaling network: evolution and hotspots of hijacking in cancer. Genome Res 22, 1222–1230 (2012). 22194470 10.1101/gr.128819.111 PMC3396364

[b7] Ubersax J. A. & Ferrell J. E. Jr. Mechanisms of specificity in protein phosphorylation. Nat Rev Mol Cell Biol 8, 530–541 (2007). 17585314 10.1038/nrm2203

[b8] Faller W. J. . mTORC1-mediated translational elongation limits intestinal tumour initiation and growth. Nature 517, 497–500 (2015). 25383520 10.1038/nature13896 PMC4304784

[b9] Martin I. . Ribosomal protein s15 phosphorylation mediates LRRK2 neurodegeneration in Parkinson’s disease. Cell 157, 472–485 (2014). 24725412 10.1016/j.cell.2014.01.064 PMC4040530

[b10] Lahiry P., Torkamani A., Schork N. J. & Hegele R. A. Kinase mutations in human disease: interpreting genotype-phenotype relationships. Nat Rev Genet 11, 60–74 (2010). 20019687 10.1038/nrg2707

[b11] Humphrey S. J., Azimifar S. B. & Mann M. High-throughput phosphoproteomics reveals *in vivo* insulin signaling dynamics. Nat Biotechnol 33, 990–995 (2015). 26280412 10.1038/nbt.3327

[b12] Zanivan S. . *In Vivo* SILAC-Based Proteomics Reveals Phosphoproteome Changes during Mouse Skin Carcinogenesis. Cell Rep 3, 552–566 (2013). 23375375 10.1016/j.celrep.2013.01.003

[b13] Lundby A. . Quantitative maps of protein phosphorylation sites across 14 different rat organs and tissues. Nat Commun 3, 876 (2012). 22673903 10.1038/ncomms1871 PMC3621391

[b14] Horn H. . KinomeXplorer: an integrated platform for kinome biology studies. Nat Methods 11, 603–604 (2014). 24874572 10.1038/nmeth.2968

[b15] Miller M. L. . Linear motif atlas for phosphorylation-dependent signaling. Sci Signal 1, ra2 (2008). 18765831 10.1126/scisignal.1159433 PMC6215708

[b16] Xue Y. . GPS 2.0, a tool to predict kinase-specific phosphorylation sites in hierarchy. Mol Cell Proteomics 7, 1598–1608 (2008). 18463090 10.1074/mcp.M700574-MCP200 PMC2528073

[b17] Qi L. . Systematic analysis of the phosphoproteome and kinase-substrate networks in the mouse testis. Mol Cell Proteomics 13, 3626–3638 (2014). 25293948 10.1074/mcp.M114.039073 PMC4256510

[b18] Beltrao P. . Systematic functional prioritization of protein posttranslational modifications. Cell 150, 413–425 (2012). 22817900 10.1016/j.cell.2012.05.036 PMC3404735

[b19] Wagih O., Reimand J. & Bader G. D. MIMP: predicting the impact of mutations on kinase-substrate phosphorylation. Nat Methods 12, 531–533 (2015). 25938373 10.1038/nmeth.3396

[b20] Wang Y. . Reconfiguring phosphorylation signaling by genetic polymorphisms affects cancer susceptibility. J Mol Cell Biol 7, 187–202 (2015). 25722345 10.1093/jmcb/mjv013

[b21] Blom N., Kreegipuu A. & Brunak S. PhosphoBase: a database of phosphorylation sites. Nucleic Acids Res 26, 382–386 (1998). 9399879 10.1093/nar/26.1.382 PMC147210

[b22] Diella F., Gould C. M., Chica C., Via A. & Gibson T. J. Phospho.ELM: a database of phosphorylation sites–update 2008. Nucleic Acids Res 36, D240–244 (2008). 17962309 10.1093/nar/gkm772 PMC2238828

[b23] Diella F. . Phospho.ELM: a database of experimentally verified phosphorylation sites in eukaryotic proteins. BMC Bioinformatics 5, 79 (2004). 15212693 10.1186/1471-2105-5-79 PMC449700

[b24] Lee T. Y. . dbPTM: an information repository of protein post-translational modification. Nucleic Acids Res 34, D622–627 (2006). 16381945 10.1093/nar/gkj083 PMC1347446

[b25] Olsen J. V. . Global, *in vivo*, and site-specific phosphorylation dynamics in signaling networks. Cell 127, 635–648 (2006). 17081983 10.1016/j.cell.2006.09.026

[b26] Gnad F., Gunawardena J. & Mann M. PHOSIDA 2011: the posttranslational modification database. Nucleic Acids Res 39, D253–260 (2011). 21081558 10.1093/nar/gkq1159 PMC3013726

[b27] Huang K. Y. . dbPTM 2016: 10-year anniversary of a resource for post-translational modification of proteins. Nucleic Acids Res (2015). 10.1093/nar/gkv1240 PMC4702878 26578568

[b28] Hornbeck P. V. . PhosphoSitePlus, 2014: mutations, PTMs and recalibrations. Nucleic Acids Res 43, D512–520 (2015). 25514926 10.1093/nar/gku1267 PMC4383998

[b29] Hornbeck P. V., Chabra I., Kornhauser J. M., Skrzypek E. & Zhang B. PhosphoSite: A bioinformatics resource dedicated to physiological protein phosphorylation. Proteomics 4, 1551–1561 (2004). 15174125 10.1002/pmic.200300772

[b30] Bodenmiller B. . PhosphoPep–a database of protein phosphorylation sites in model organisms. Nat Biotechnol 26, 1339–1340 (2008). 19060867 10.1038/nbt1208-1339 PMC2743685

[b31] Bodenmiller B. . PhosphoPep–a phosphoproteome resource for systems biology research in Drosophila Kc167 cells. Mol Syst Biol 3, 139 (2007). 17940529 10.1038/msb4100182 PMC2063582

[b32] Sadowski I. . The PhosphoGRID Saccharomyces cerevisiae protein phosphorylation site database: version 2.0 update. Database (Oxford) 2013, bat026 (2013). 23674503 10.1093/database/bat026 PMC3653121

[b33] Stark C. . PhosphoGRID: a database of experimentally verified *in vivo* protein phosphorylation sites from the budding yeast Saccharomyces cerevisiae. Database (Oxford) 2010, bap026 (2010). 20428315 10.1093/database/bap026 PMC2860897

[b34] Li J. . SysPTM 2.0: an updated systematic resource for post-translational modification. Database (Oxford) 2014, bau025 (2014). 24705204 10.1093/database/bau025 PMC3975108

[b35] Li H. . SysPTM: a systematic resource for proteomic research on post-translational modifications. Mol Cell Proteomics 8, 1839–1849 (2009). 19366988 10.1074/mcp.M900030-MCP200 PMC2722767

[b36] Goel R., Harsha H. C., Pandey A. & Prasad T. S. Human Protein Reference Database and Human Proteinpedia as resources for phosphoproteome analysis. Mol Biosyst 8, 453–463 (2012). 22159132 10.1039/c1mb05340j PMC3804167

[b37] UniProt: a hub for protein information. Nucleic Acids Res 43, D204–212 (2015). 25348405 10.1093/nar/gku989 PMC4384041

[b38] Boratyn G. M. . BLAST: a more efficient report with usability improvements. Nucleic Acids Res 41, W29–33 (2013). 23609542 10.1093/nar/gkt282 PMC3692093

[b39] Hunter T. Tyrosine phosphorylation: thirty years and counting. Curr Opin Cell Biol 21, 140–146 (2009). 19269802 10.1016/j.ceb.2009.01.028 PMC2670436

[b40] Wang Y. . EKPD: a hierarchical database of eukaryotic protein kinases and protein phosphatases. Nucleic Acids Res 42, D496–502 (2014). 24214991 10.1093/nar/gkt1121 PMC3965077

[b41] Lindberg R. A., Quinn A. M. & Hunter T. Dual-specificity protein kinases: will any hydroxyl do? Trends Biochem Sci 17, 114–119 (1992). 1412695 10.1016/0968-0004(92)90248-8

[b42] Iakoucheva L. M. . The importance of intrinsic disorder for protein phosphorylation. Nucleic Acids Res 32, 1037–1049 (2004). 14960716 10.1093/nar/gkh253 PMC373391

[b43] Minguez P. . Deciphering a global network of functionally associated post-translational modifications. Mol Syst Biol 8, 599 (2012). 22806145 10.1038/msb.2012.31 PMC3421446

[b44] van Wijk K. J., Friso G., Walther D. & Schulze W. X. Meta-Analysis of Arabidopsis thaliana Phospho-Proteomics Data Reveals Compartmentalization of Phosphorylation Motifs. Plant Cell 26, 2367–2389 (2014). 24894044 10.1105/tpc.114.125815 PMC4114939

[b45] Schwartz D. & Gygi S. P. An iterative statistical approach to the identification of protein phosphorylation motifs from large-scale data sets. Nat Biotechnol 23, 1391–1398 (2005). 16273072 10.1038/nbt1146

[b46] Sonnhammer E. L. & Ostlund G. InParanoid 8: orthology analysis between 273 proteomes, mostly eukaryotic. Nucleic Acids Res 43, D234–239 (2015). 25429972 10.1093/nar/gku1203 PMC4383983

[b47] Sievers F. . Fast, scalable generation of high-quality protein multiple sequence alignments using Clustal Omega. Mol Syst Biol 7, 539 (2011). 21988835 10.1038/msb.2011.75 PMC3261699

[b48] Letunic I. & Bork P. Interactive Tree Of Life v2: online annotation and display of phylogenetic trees made easy. Nucleic Acids Res 39, W475–478 (2011). 21470960 10.1093/nar/gkr201 PMC3125724

