# Supplementary material for: dbPAF: an integrative database of protein phosphorylation in animals and fungi
Source: Sci Rep. 2016 Mar 24;6:23534. doi: 10.1038/srep23534 (PMC4806352; doi:10.1038/srep23534)
Supplement: Supplementary Information [file srep23534-s1.pdf]

Supplementary Information

dbPAF: an integrative database of protein phosphorylation in animals and fungi

Shahid Ullah[1,†], Shaofeng Lin[1,†], Yang Xu[1], Wankun Deng[1], Lili Ma[1], Ying Zhang[1], Zexian Liu[1,*], and Yu Xue[1,2,*]

[1]Department of Bioinformatics & Systems Biology, College of Life Science and Technology, Huazhong University of Science and Technology, Wuhan, Hubei 430074, China

[2]Key Laboratory of Molecular Biophysics of the Ministry of Education, College of Life Science and Technology, Huazhong University of Science and Technology, Wuhan, Hubei 430074, China

[†]These authors contributed equally to this work.

[*]Correspondence and requests for materials should be addressed to Y.Xue. (xueyu@hust.edu.cn) or Z.L. (lzx@hust.edu.cn).

Supplementary Table S1 - From the scientific literature, we totally collected 294,370 non-redundant phosphorylation sites of 40,432 protein substrates identified from large-scale phosphoproteomic studies. *a*. The pubmed ID of original reference; *b*. The number of proteins; *c* . The number of phosphorylation sites.

PMID[a]	Num. of proteins[b]	Num. of sites[c]
26447709	495	928
26270265	24	47
26267517	288	700
26160508	415	587
26074081	4731	18331
26040406	1267	3949
26040289	1910	7630
26029660	22	30
25882841	415	784
25852190	201	287
25850435	4723	17502
25849741	2731	7193
25704821	501	1139
25627689	6972	49761
25532521	613	1309
25521595	2637	6869
25492886	13	41
25458152	26	30
25404012	466	610
25348954	58	73
25348772	162	235
25338131	6006	35395
25315811	2212	12086
25266776	3508	15736
25262027	1900	4110
25219547	332	712
25195567	410	574
25162660	41	43
25142963	15	21
25003641	1065	1608
24972180	509	701
24961812	2067	8916
24961811	4038	14763
24945867	393	1022
24888630	43	62
24825855	5	5
24769528	66	257
24732914	904	2760
24723360	795	1337
24708550	30	44
24704852	164	268
24702127	883	1374
24670416	29	49
24667141	1147	2824

24603354	223	321
24511133	50	64
24400094	304	637
24260401	841	1204
24129246	84	103
24117733	1141	1934
23984901	6989	14743
23917254	3603	11806
23911959	3071	7127
23909892	50	85
23898821	4084	15867
23882029	257	378
23882026	230	391
23836654	704	1461
23832136	333	483
23825934	338	604
23800682	409	769
23712012	1743	7089
23684622	2888	10868
23663014	2443	7531
23607784	407	739
23589303	127	178
23567750	1057	1668
23527152	633	980
23403867	1349	2886
23384938	329	393
23353032	90	104
23186163	3870	9539
23090842	2712	10401
22997577	5	5
22923814	78	126
22817900	22096	112587
22807455	1669	5022
22798277	965	1536
22369663	1945	8033
22276854	1323	2550
22199227	2407	5996
22135298	190	248
22115753	1097	2275
21955146	58	73
21857030	927	1441
21743459	2848	8102
21630457	2082	9224
21609022	48	86
21373199	80	98
21183079	6321	35205
20873877	2720	9687
20833797	64	120
20688971	36	58
20469934	949	1849
20377248	1258	5184

20363803	1187	2949
20071362	439	819
20068231	4917	25042
20047950	341	834
19823750	1604	5590
19795423	725	1856
19691289	210	401
19690332	2771	10318
19547744	736	1347
19369195	175	492
18846507	1219	2488
18806794	2441	7577
18779572	317	547
18691976	529	1393
18578522	119	222
18522436	7	13
18407956	1961	5780
18212344	106	126
17287358	384	692
16445868	160	337
Total	40432	294370
